# Assessing the impact of alcohol consumption on the genetic contribution to mean corpuscular volume

**DOI:** 10.1093/hmg/ddab147

**Published:** 2021-06-08

**Authors:** Andrew Thompson, Katharine King, Andrew P Morris, Munir Pirmohamed

**Affiliations:** Wolfson Centre for Personalised Medicine, Institute of Systems, Molecular and Integrative Biology, University of Liverpool, Liverpool L69 3GL, UK; MRC Centre for Drug Safety Science, Institute of Systems, Molecular and Integrative Biology, University of Liverpool, Liverpool L69 3GL, UK; Liverpool Centre for Alcohol Research, University of Liverpool, Liverpool L69 3BX, UK; Wolfson Centre for Personalised Medicine, Institute of Systems, Molecular and Integrative Biology, University of Liverpool, Liverpool L69 3GL, UK; MRC Centre for Drug Safety Science, Institute of Systems, Molecular and Integrative Biology, University of Liverpool, Liverpool L69 3GL, UK; Division of Cardiovascular Sciences, Faculty of Biology, Medicine and Health, The University of Manchester, Manchester M13 9NT, UK; Division of Musculoskeletal and Dermatological Sciences, Centre for Genetics and Genomics Versus Arthritis, Centre for Musculoskeletal Research, The University of Manchester, Manchester M13 9PL, UK; Department of Biostatistics, University of Liverpool, Liverpool L69 3GL, UK; Wolfson Centre for Personalised Medicine, Institute of Systems, Molecular and Integrative Biology, University of Liverpool, Liverpool L69 3GL, UK; MRC Centre for Drug Safety Science, Institute of Systems, Molecular and Integrative Biology, University of Liverpool, Liverpool L69 3GL, UK; Liverpool Centre for Alcohol Research, University of Liverpool, Liverpool L69 3BX, UK; Liverpool University Hospital, Liverpool L9 7AL, UK; Liverpool Health Partners, Liverpool L3 5TF, UK

## Abstract

The relationship between the genetic loci that influence mean corpuscular volume (MCV) and those associated with excess alcohol drinking is unknown. We used white British participants from the UK Biobank (*n* = 362 595) to assess the association between alcohol consumption and MCV, and whether this was modulated by genetic factors. Multivariable regression was applied to identify predictors of MCV. GWAS, with and without stratification for alcohol consumption, determined how genetic variants influence MCV. SNPs in *ADH1B*, *ADH1C* and *ALDH1B* were used to construct a genetic score to test the assumption that acetaldehyde formation is an important determinant of MCV. Additional investigations using Mendelian randomization and phenome­wide association analysis were conducted. Increasing alcohol consumption by 40 g/week resulted in a 0.30% [95% confidence interval CI: 0.30–0.31%] increase in MCV (*P* < 1.0 × 10^−320^). Unstratified (irrespective of alcohol intake) GWAS identified 212 loci associated with MCV, of which 108 were novel. There was no heterogeneity of allelic effects by drinking status. No association was found between MCV and the genetic score generated from alcohol metabolizing genes. Mendelian randomization demonstrated a causal effect for alcohol on MCV. Seventy-one SNP-outcome pairs reached statistical significance in phenome­wide association analysis, with evidence of shared genetic architecture for MCV and thyroid dysfunction, and mineral metabolism disorders. MCV increases linearly with alcohol intake in a causal manner. Many genetic loci influence MCV, with new loci identified in this analysis that provide novel biological insights. However, there was no interaction between alcohol consumption and the allelic variants associated with MCV.

## Introduction

Alcohol misuse and abuse is a leading cause of morbidity and mortality (1). In 2016, global statistics suggested that 5.1% (~3 million) of deaths and 5.3% (~133 million) of disability-adjusted life years were caused by the harmful use of alcohol (2). Early identification of individuals who are misusing alcohol is critical for interventions to stop progression towards alcohol dependence and alcohol-related end-organ damage. Unfortunately, a history from a patient is not always reliable, and laboratory tests vary in their diagnostic accuracy, availability and usage. In clinical practice, therefore, it is usual to use a combination of history (including alcohol intake and symptoms consistent with organ damage), physical examination (to look for features of organ damage) and laboratory markers that support alcohol misuse as the underlying aetiology. The most widely used laboratory tests are (a) liver function tests (in particular, gamma-glutamyl transferase), which indicates liver damage, and (b) the mean corpuscular volume (MCV), a measure of the mean size and volume of erythrocytes, which is a non-specific marker of alcohol misuse (3).

The molecular basis for the increase in MCV that occurs with alcohol misuse is incompletely understood. A study of 105 alcohol-dependent individuals, 62 moderate drinkers and 24 abstainers was able to show that the increase in MCV was dose-dependent (4). Alcohol may have direct haematotoxic effects by interfering with cell structure and erythrocyte stability (5). Interestingly, the levels of acetaldehyde, a metabolite of alcohol, show a significant increase inside erythrocytes of alcohol-dependent individuals (6). Since acetaldehyde is a toxic metabolite, and can bind to proteins, this may lead to erythrocyte damage directly or through an immune-mediated mechanism via the development of anti-acetaldehyde adduct antibodies (4). Folate deficiency also occurs with alcohol misuse, particularly in those patients with liver disease (7), and therefore may be implicated in increasing the MCV (8).

It is important to note, however, that MCV is a non-specific biomarker in that other factors such as age, smoking, malnutrition and underlying diseases, including hypothyroidism, liver disease and pernicious anaemia, are also known to affect MCV (7,9,10). There is also a genetic contribution to the MCV, in addition to the genetic factors that have been identified for other haematological indices (11,12). Genetic factors have also been implicated in high alcohol consumption—our recent study showed genome-wide significant effects across six loci following meta-analysis in two large independent cohorts (13). The relationship between the genetic loci that determine the MCV and those associated with excess alcohol drinking is, however, unknown.

In this study, we have used genome-wide association study (GWAS) data from the UK Biobank (UKB) to understand if genetic variants and level of alcohol consumption interact to influence MCV. The specific aims were as follows: (1) determine how genetic loci influence MCV, with and without stratification for alcohol consumption; (2) confirm the causal effect of alcohol consumption on MCV using Mendelian randomization and (3) explore the association between acetaldehyde accumulation and MCV using genotype data from alcohol metabolizing genes.

## Results

### Demographics

A total of 139 921 individuals were excluded from the UKB cohort: 1502 declined to provide information on their drinking status, 1984 were drinking at levels at least 4 SDs above sex-specific means, 7060 had liver disease, 23 385 had missing MCV data, 104 788 failed GWAS quality control (including ethnic inclusion) and 1202 had missing covariate information. This study therefore included 362 595 participants, of whom 194 706 (53.7%) were females and the average age was 56.9 (SD = 7.9) years. There were 82 235 (24.6%) zero, 146 436 (40.1%) light, 114 946 (30.2%) moderate and 18 978 (5.0%) heavy drinkers, with the median units/week being 6.0 [interquartile (IQR) = 13.0] in females and 15.6 (IQR = 23.9) in males. There was evidence of hypothyroidism and vitamin B12 deficiency in 14 781 and 777 participants, respectively.

### Alcohol consumption and MCV

Alcohol consumption was associated with higher MCV (*P* < 1.0 × 10^−320^). Increasing alcohol consumption by 5 units (40 g) per week resulted in a 0.3% increase in MCV. Variation by drinking status was evident; compared with light drinkers (reference group), zero drinkers had 0.9% lower mean values, while moderate and heavy drinkers had 1.1 and 2.8% higher mean values, respectively (all *P* < 1.0 × 10^−320^; Table 1). Results from multivariate analysis for MCV were consistent in terms of direction and magnitude when those classified as teetotal were removed (Supplementary Material Table S1).

**Table 1:** Summary of linear and logistic regression models with all participants (*n* = 362 595)

Risk factor		Change in MCV (%)	95% CI	*P*
Alcohol: continuous variable				
Alcohol (5 units)		0.30	0.30 to 0.31	<1.0 × 10^−320^
Sex (Ref: female)		−0.21	−0.25 to −0.18	5.2 × 10^−38^
Age at recruitment		0.06	0.06 to 0.06	<1.0 × 10^−320^
Never smoker (Ref: current)		−2.00	−2.05 to −1.95	<1.0 × 10^−320^
Previous smoker (Ref: current)		−1.87	−1.93 to −1.82	<1.0 × 10^−320^
Hypothyroidism		−0.20	−0.28 to −0.13	2.4 × 10^−7^
B12 deficiency		0.20	−0.13 to 0.53	0.23
				
Alcohol: categorical variable				
Drinking status (Ref: light) Non-drinker Moderate Heavy		−0.89 1.14 2.80	−0.93 to −0.85 1.10 to 1.17 2.72 to 2.87	< 1.0 × 10^−320^ <1.0 × 10^−320^ <1.0 × 10^−320^
Sex (Ref: female)		−0.08	−0.11 to −0.05	1.9 × 10^−6^
Age at recruitment		0.06	0.06 to 0.07	<1.0 × 10^−320^
Never smoker (Ref: current)		−2.11	−2.16 to −2.06	<1.0 × 10^−320^
Previous smoker (Ref: current)		−1.97	−2.02 to −1.92	<1.0 × 10^−320^
Hypothyroidism		−0.17	−0.25 to −0.09	1.4 × 10^−5^
B12 deficiency		0.26	−0.06 to 0.59	0.12

### GWAS of MCV

**Figure 1:**
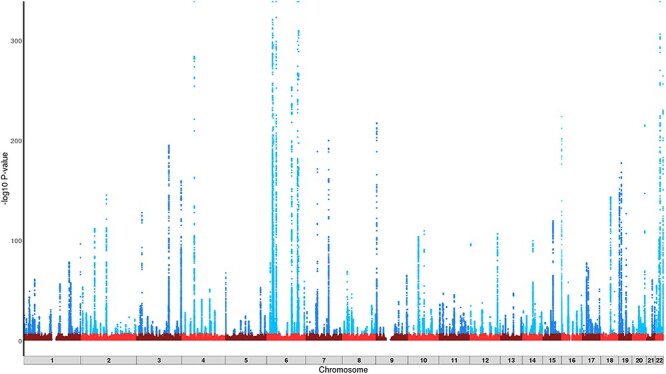
Manhattan plot of the unstratified GWAS outcomes for MCV (*n* = 362 595). *Y* axis truncated at 1 × 10^−320^.

**Figure 2:**
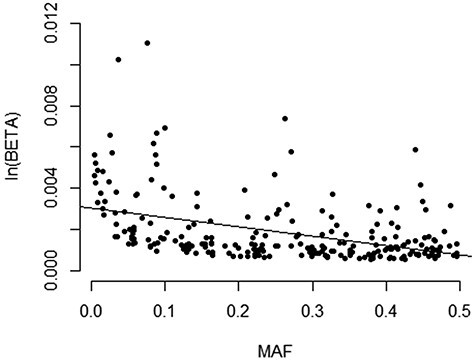
Relationship between minor allele frequency and effect size.

An unstratified (i.e. irrespective of alcohol intake) GWAS in white British individuals identified 212 loci associated with MCV at *P* < 5 × 10^−8^ (Fig. 1). Presented *P*-values are corrected based on a LD score regression intercept = 1.20. The large sample size (*n* = 362 595) resulted in identification of variants with small effect sizes, equivalent to a change in MCV of 0.057% (Table 2). There was evidence to suggest that lower minor allele frequency was associated with larger effect sizes (Fig. 2). The largest effect size was observed with rs144861591 [effect allele frequency (EAF) 0.076; *P* = 3.4 × 10^−640^], where the minor allele (T) was associated with an increase in MCV by 1.11%. This variant is located ~13.5 bp downstream of LOC108783645, an *HFE* antisense RNA. *HFE* itself is involved with iron regulation and has been associated with haemochromatosis (14). Strong associations were also reported in loci mapping to *HBS1L-MYB, TMPRSS6, CCND3, CARMIL1, ODF3B* and *CCDC162P* (11,12,15–18). We compared (using SNP ID and reported gene symbol) our findings to those of other equivalently sized genome-wide studies of MCV in UKB (11,19) and found that 58.0% (*n* = 123) of our loci were unique, likely due to targeted study of MCV and trait-specific covariate control whereas the cited studies explored multiple traits. Investigation in the GWAS catalog (https://www.ebi.ac.uk/gwas/) found replication with a further 15 mapped genes, leaving 108 new findings (Table 2). The SNP-based heritability of MCV was estimated to be 24.2% through LD score regression.

**Table 2:** Summary of genome-wide significant SNPs following distance-based clumping

SNP	CHR	BP	Locus (overlapping/nearest)	Effect allele	EAF	% change MCV	95%LCI	%UCI	*P* (GC corrected)	Unique^a^
rs7775698	6	135 418 635	HBS1L	C	0.738	--0.73	--0.76	--0.71	2.8e−790	N
rs144861591	6	26 072 992	HFE	C	0.924	--1.10	--1.14	--1.06	3.4e−640	N
rs855791	22	37 462 936	TMPRSS6	A	0.440	--0.59	--0.61	--0.56	3.0e−610	N
rs9471708	6	41 956 353	CCND3	C	0.728	0.58	0.56	0.60	1.3e−493	N
rs592423	6	139 840 693	RP11-12A2.3	A	0.447	0.42	0.40	0.44	2.2e−327	N
rs149359690	6	25 526 319	LRRC16A	C	0.900	--0.69	--0.72	--0.65	3.6e−325	N
rs218264	4	55 408 875	AC006552.1	A	0.752	--0.46	--0.49	--0.44	8.7E−287	Y
rs13191659	6	27 001 055	VN1R12P	C	0.911	--0.66	--0.70	--0.63	1.7E−264	Y
rs140522	22	50 971 266	ODF3B	T	0.327	--0.37	--0.39	--0.35	1.4E−221	N
rs9487023	6	109 590 004	C6orf183	A	0.551	--0.34	--0.36	--0.31	3.6E−212	N
rs71559031	6	27 519 947	XXbac-BPG34I8.3	G	0.916	--0.62	--0.66	--0.58	2.7E−203	Y
rs117747069	16	170 076	NPRL3	G	0.963	1.03	0.96	1.10	9.1E−188	N
rs10758657	9	4 853 751	RCL1	A	0.791	0.39	0.37	0.42	1.8E−182	N
rs6014993	20	55 991 637	RBM38	A	0.513	--0.32	--0.34	--0.30	1.2E−180	N
rs147493146	6	28 054 465	ZNF165	C	0.913	--0.56	--0.60	--0.52	5.2E−179	Y
rs9801017	7	100 236 202	TFR2	G	0.376	--0.31	--0.34	--0.29	5.7E−168	N
rs7428496	3	142 320 532	PLS1	A	0.414	0.30	0.28	0.33	7.4E−164	N
rs6592965	7	50 427 982	IKZF1	G	0.546	--0.30	--0.32	--0.28	1.3E−158	N
rs8110787	19	12 999 458	KLF1	C	0.609	--0.29	--0.31	--0.27	3.2E−149	N
rs114179634	6	28 626 101	LINC00533	C	0.911	--0.51	--0.55	--0.47	2.3E−146	Y
rs13194984	6	26 500 563	BTN1A1	G	0.856	--0.38	--0.41	--0.35	1.7E−134	N
rs41298087	3	195 779 736	TFRC	C	0.687	0.29	0.27	0.32	4.0E−134	N
rs1045267	2	112 187 041	NA	A	0.266	0.32	0.29	0.35	2.8E−122	N
rs9952469	18	43 812 010	C18orf25	T	0.254	0.30	0.27	0.32	9.1E−121	N
rs362538	6	29 510 630	GPR53P	G	0.902	--0.40	--0.44	--0.36	2.4E−109	Y
rs113700287	3	24 334 511	THRB	T	0.326	--0.26	--0.28	--0.24	1.1E−107	N
rs78744187	19	33 754 548	CTD-2540B15.12	C	0.918	0.44	0.40	0.48	7.7E−107	N
rs113968785	15	65 734 015	DPP8	A	0.748	0.28	0.25	0.30	4.3E−101	Y
rs243076	2	60 617 563	AC007381.2	G	0.594	--0.23	--0.25	--0.21	1.9E−94	Y
rs17476364	10	71 094 504	HK1	T	0.890	--0.36	--0.39	--0.32	1.5E−92	N
rs2057726	6	30 335 350	UBQLN1P1	C	0.143	0.31	0.28	0.34	1.5E−91	Y
rs56273049	12	121 156 041	UNC119B	A	0.612	0.23	0.21	0.25	4.3E−90	N
rs7089063	10	45 946 389	RP11-67C2.2	C	0.761	--0.26	--0.28	--0.23	8.0E−88	Y
rs738264	22	32 874 258	FBXO7	G	0.728	0.24	0.22	0.27	1.7E−85	N
rs536350318	12	4 331 068	CCND2-AS1	C	0.788	--0.26	--0.28	--0.23	1.1E−81	N
rs3811444	1	248 039 451	TRIM58	C	0.667	0.22	0.20	0.24	1.1E−81	N
rs2026428	10	45 389 452	TMEM72-AS1	T	0.416	--0.21	--0.23	--0.19	3.3E−75	N
rs16843346	1	198 543 027	RP11-553K8.2	C	0.972	--0.57	--0.64	--0.51	1.7E−66	Y
rs1042391	6	16 290 761	GMPR	T	0.619	0.19	0.17	0.21	9.5E−66	N
rs56142708	17	19 934 963	SPECC1	A	0.478	--0.19	--0.21	--0.17	2.8E−65	N
rs12193223	6	24 978 511	FAM65B	C	0.938	--0.37	--0.42	--0.33	1.3E−62	Y
rs17296501	8	21 849 566	XPO7	C	0.838	--0.24	--0.27	--0.21	8.1E−59	N
rs7705526	5	1 285 974	TERT	C	0.674	0.19	0.17	0.21	1.6E−57	N
rs10901252	9	136 128 000	ABO	G	0.940	0.36	0.32	0.41	2.5E−55	N
rs74401481	19	1 855 583	KLF16	C	0.975	0.66	0.57	0.74	3.5E−55	N
rs13207150	6	110 092 900	FIG4	C	0.313	--0.18	--0.20	--0.15	1.2E−52	N
rs4134058	1	47 670 911	TAL1	T	0.457	--0.17	--0.19	--0.15	5.4E−52	N
rs2834256	21	35 125 373	AP000304.12	A	0.655	0.18	0.15	0.20	1.4E−51	N
rs381500	6	164 478 388	RP1-155D22.2	C	0.549	0.16	0.14	0.18	2.5E−50	N
rs1172113	1	205 226 288	TMCC2	T	0.587	--0.16	--0.19	--0.14	3.0E−49	N
rs12128171	1	158 580 477	SPTA1	A	0.719	0.18	0.15	0.20	3.3E−48	N
rs6730558	2	8 756 183	AC011747.6	C	0.620	0.16	0.14	0.18	9.6E−46	N
rs111918234	5	154 027 482	MIR1303	GT	0.860	--0.23	--0.26	--0.19	3.1E−45	N
rs558567978	17	76 124 810	TMC6	A	0.777	0.19	0.16	0.21	6.8E−44	N
rs67775544	4	122 796 917	RP11-63B13.1	G	0.603	--0.16	--0.18	--0.13	8.4E−44	Y
rs17534202	1	203 281 175	BTG2	G	0.464	0.15	0.13	0.17	5.9E−42	N
rs111721712	6	31 315 407	HLA-B	C	0.534	0.15	0.13	0.17	8.5E−42	N
rs10766533	11	19 224 677	CSRP3	T	0.278	0.16	0.14	0.19	1.7E−40	N
rs11227793	11	67 194 593	RPS6KB2	C	0.561	0.15	0.12	0.17	3.4E−39	Y
rs67971539	16	87 886 726	SLC7A5	T	0.764	--0.17	--0.19	--0.14	8.6E−39	N
rs4134898	7	99 711 614	TAF6	C	0.868	0.21	0.18	0.24	1.7E−37	Y
rs17776547	10	51 581 143	NCOA4	A	0.965	--0.38	--0.44	--0.32	4.3E−36	N
rs1567668	8	23 418 122	SLC25A37	A	0.429	--0.14	--0.16	--0.12	1.6E−35	N
rs144894688	9	100 746 855	ANP32B	G	0.780	0.16	0.13	0.19	1.6E−33	N
rs35979828	12	54 685 880	RP11-968A15.8	C	0.931	--0.26	--0.30	--0.22	1.8E−32	N
rs114165892	14	74 223 968	ELMSAN1	C	0.976	--0.43	--0.50	--0.36	6.1E−32	Y
rs7789162	7	44 872 900	H2AFV	T	0.495	--0.13	--0.15	--0.11	1.5E−31	N
rs145946844	2	111 612 050	ACOXL	T	0.662	--0.13	--0.16	--0.11	7.1E−31	N
rs59027521	8	128 966 573	PVT1	A	0.657	0.13	0.11	0.16	2.4E−30	N
rs6440006	3	141 142 691	ZBTB38	G	0.552	0.13	0.11	0.15	3.0E−30	Y
rs6788010	3	16 929 109	PLCL2	T	0.920	0.23	0.19	0.27	9.1E−30	N
rs496321	11	94 886 632	RP11-712B9.2	T	0.386	0.13	0.10	0.15	1.3E−28	N
rs1991866	8	130 624 105	CCDC26	G	0.424	0.12	0.10	0.15	1.7E−28	N
rs592229	6	31 930 441	SKIV2L	G	0.486	0.12	0.10	0.14	1.9E−28	Y
rs10883359	10	101 274 033	RP11-129J12.2	A	0.716	--0.13	--0.16	--0.11	5.0E−27	Y
rs10923397	1	118 251 143	RP11-134N8.2	C	0.837	--0.16	--0.19	--0.13	1.1E−26	N
rs78147199	15	66 245 962	MEGF11	G	0.955	0.29	0.23	0.34	1.2E−26	Y
rs12582170	12	53 757 831	SP1	A	0.156	--0.16	--0.19	--0.13	1.4E−26	N
rs2713936	15	56 545 985	TEX9	A	0.577	0.12	0.10	0.14	3.8E−26	N
rs11723371	4	145 025 810	RP11-673E1.4	T	0.527	--0.12	--0.14	--0.10	4.3E−26	N
rs151305716	20	52 222 106	ZNF217	C	0.984	--0.48	--0.57	--0.39	4.9E−26	N
rs111836360	16	89 786 649	VPS9D1	A	0.594	--0.12	--0.14	--0.09	4.7E−25	N
rs2277339	12	57 146 069	PRIM1	T	0.897	--0.19	--0.22	--0.15	6.6E−25	N
rs4900538	14	102 994 065	MIR4309	T	0.355	--0.12	--0.14	--0.10	9.0E−25	N
rs1134634	4	15 603 069	CC2D2A	G	0.414	--0.11	--0.14	--0.09	1.9E−24	N
rs136211	22	36 758 547	MYH9	A	0.313	0.12	0.10	0.14	3.9E−24	N
rs371182872	11	108 300 854	C11orf65	CT	0.588	0.11	0.09	0.14	7.7E−24	N
rs322918	1	199 068 614	RP11-16L9.4	A	0.539	0.11	0.09	0.13	8.0E−23	N
rs1569419	1	2 996 602	PRDM16	T	0.233	0.13	0.10	0.15	4.2E−22	N
rs9532563	13	41 160 270	FOXO1	T	0.782	0.13	0.10	0.16	4.8E−22	N
rs200234036	1	39 869 512	MACF1	GC	0.728	0.12	0.10	0.15	7.1E−22	Y
rs875742	5	173 287 763	CPEB4	G	0.594	0.11	0.09	0.13	7.3E−22	N
rs9370792	6	15 099 585	RP1-190J20.2	A	0.601	--0.11	--0.13	--0.08	1.1E−21	Y
rs138191091	7	99 105 676	ZKSCAN5	A	0.877	0.17	0.13	0.20	1.3E−21	Y
rs4955426	3	49 143 438	QARS	C	0.222	0.12	0.10	0.15	4.2E−21	Y
rs2143583	1	114 989 211	TRIM33	T	0.747	--0.12	--0.14	--0.09	4.2E−21	N
rs10883710	10	103 885 557	LDB1	T	0.545	--0.10	--0.13	--0.08	5.9E−21	Y
rs7487314	12	88 836 215	Y_RNA	G	0.299	--0.11	--0.14	--0.09	6.0E−21	N
rs35362007	14	96 003 198	GLRX5	G	0.749	--0.12	--0.15	--0.10	7.8E−21	N
rs632959	1	68 197 671	GNG12	A	0.295	--0.11	--0.14	--0.09	9.2E−21	N
rs1047891	2	211 540 507	CPS1	C	0.684	--0.11	--0.13	--0.09	1.1E−20	N
rs2923403	8	42 447 748	RP11-503E24.3	G	0.407	0.10	0.08	0.13	2.2E−20	Y
rs2224539	20	38 552 107	HSPE1P1	A	0.584	--0.10	--0.13	--0.08	3.5E−20	N
rs149472343	10	64 905 218	NRBF2	C	0.967	0.28	0.22	0.34	1.3E−19	N
rs2237572	7	92 260 260	CDK6	T	0.804	--0.12	--0.15	--0.10	2.9E−19	N
rs545709142	1	11 881 141	CLCN6	C	0.837	0.13	0.10	0.16	3.3E−19	N
rs12779263	10	104 886 533	NT5C2	G	0.706	0.11	0.08	0.13	3.6E−19	Y
rs322351	5	172 194 873	RP11-779O18.3	C	0.532	--0.10	--0.12	--0.08	4.5E−19	Y
rs72766638	9	136 931 778	BRD3	C	0.836	--0.13	--0.16	--0.10	6.1E−19	Y
rs174567	11	61 593 005	FADS2	A	0.649	0.10	0.08	0.12	8.3E−19	Y
rs4663199	2	236 368 039	AGAP1	T	0.601	--0.10	--0.12	--0.08	1.7E−18	N
rs74929147	19	18 413 061	LSM4	G	0.941	0.21	0.16	0.26	2.2E−18	N
rs201581170	10	105 682 344	OBFC1	T	0.845	0.13	0.10	0.16	3.2E−18	Y
rs9866749	3	49 650 935	BSN	A	0.296	0.11	0.08	0.13	4.7E−18	Y
rs2492301	1	37 939 173	LINC01137	T	0.471	0.10	0.07	0.12	6.0E−18	N
rs80226431	8	48 267 917	SPIDR	A	0.905	0.16	0.12	0.20	2.0E−17	Y
rs36225153	4	146 081 852	OTUD4	C	0.886	0.15	0.11	0.18	2.8E−17	Y
rs7641761	3	178 740 422	ZMAT3	T	0.302	--0.10	--0.12	--0.08	3.1E−17	N
rs6116019	20	3 742 066	C20orf27	T	0.901	--0.16	--0.19	--0.12	3.7E−17	N
rs4936291	11	114 009 982	ZBTB16	A	0.611	0.10	0.07	0.12	4.0E−17	Y
rs6531706	4	39 296 167	RFC1	T	0.565	--0.09	--0.12	--0.07	5.8E−17	N
rs73079476	12	21 343 833	SLCO1B1	A	0.850	0.13	0.10	0.16	6.1E−17	Y
rs79953286	3	132 226 100	DNAJC13	A	0.942	0.20	0.15	0.24	8.3E−17	Y
rs1867146	15	75 354 971	PPCDC	C	0.818	0.12	0.09	0.15	9.0E−17	Y
rs2723513	7	17 814 888	SNX13	A	0.461	--0.09	--0.11	--0.07	1.2E−16	Y
rs1119279	8	47 071 977	AC113134.1	C	0.903	0.15	0.12	0.19	1.9E−16	Y
rs145498761	4	128 456 129	RP11-18O11.2	T	0.991	0.49	0.37	0.61	3.7E−16	Y
rs920112	2	174 219 135	AC092573.2	G	0.947	--0.20	--0.25	--0.15	4.7E−16	N
rs2134814	6	90 987 512	BACH2	C	0.646	--0.09	--0.11	--0.07	5.4E−16	N
rs12196049	6	121 786 091	RNU4-35P	A	0.801	0.11	0.08	0.14	6.7E−16	Y
rs964184	11	116 648 917	ZNF259	G	0.132	--0.13	--0.16	--0.10	6.7E−16	Y
rs13209786	6	131 421 040	AKAP7	A	0.769	0.10	0.08	0.13	8.0E−16	Y
rs145185045	17	60 091 881	MED13	T	0.770	0.11	0.08	0.13	1.4E−15	Y
rs3892355	19	5 696 962	LONP1	G	0.674	--0.09	--0.12	--0.07	1.9E−15	Y
rs61952071	12	133 076 439	FBRSL1	C	0.687	--0.09	--0.12	--0.07	2.0E−15	Y
rs6711700	2	86 987 987	RMND5A	G	0.329	--0.09	--0.12	--0.07	2.2E−15	N
rs147707926	9	115 914 583	SLC31A2	C	0.981	0.34	0.25	0.42	2.6E−15	Y
rs4672497	2	62 523 565	snoU13	C	0.779	0.10	0.08	0.13	5.0E−15	N
rs657036	6	35 901 151	SLC26A8	G	0.703	0.09	0.07	0.12	5.0E−15	Y
rs7137095	12	6 739 907	LPAR5	C	0.452	--0.09	--0.11	--0.07	8.3E−15	Y
rs1330826	9	85 129 970	RP11-15B24.5	G	0.772	--0.10	--0.13	--0.08	1.2E−14	N
rs139012450	2	160 684 717	LY75	T	0.504	--0.09	--0.11	--0.06	1.3E−14	Y
rs75497126	3	141 655 542	RP11-271K21.11	A	0.993	0.52	0.39	0.66	2.8E−14	Y
rs200401106	2	197 024 922	STK17B	A	0.871	0.12	0.09	0.16	4.2E−14	Y
rs75581061	6	134 858 499	RP11-557H15.4	A	0.877	--0.12	--0.16	--0.09	7.9E−14	Y
rs62472014	7	98 517 117	TRRAP	C	0.966	0.23	0.17	0.29	8.8E−14	N
rs184837332	17	44 359 783	ARL17B	G	0.775	0.10	0.07	0.13	9.4E-14	Y
rs78378222	17	7 571 752	TP53	T	0.987	0.38	0.28	0.48	9.9E−14	Y
rs6747952	2	239 069 926	FAM132B	C	0.556	--0.08	--0.10	--0.06	1.3E−13	Y
rs10865309	2	58 984 870	LINC01122	C	0.863	0.12	0.09	0.15	1.3E−13	N
rs117325033	6	140 511 519	MIR3668	T	0.995	--0.56	--0.71	--0.41	1.4E−13	Y
rs115447786	6	34 354 073	NUDT3	C	0.955	0.19	0.14	0.24	1.6E−13	Y
rs4285804	10	104 386 309	SUFU	T	0.441	0.08	0.06	0.10	2.2E−13	Y
rs1958078	14	70 354 858	SMOC1	A	0.155	--0.11	--0.14	--0.08	2.5E−13	N
rs35158985	16	68 796 746	CDH1	A	0.693	0.09	0.06	0.11	4.1E−13	N
rs2023335	19	10 695 959	AP1M2	A	0.056	--0.18	--0.23	--0.13	4.2E−13	Y
rs10893817	11	127 951 980	RP11-702B10.2	A	0.351	--0.08	--0.11	--0.06	4.9E−13	Y
rs766009815	3	176 878 261	TBL1XR1	CA	0.432	--0.08	--0.10	--0.06	5.3E−13	Y
rs34406510	1	209 936 964	TRAF3IP3	T	0.766	0.09	0.07	0.12	7.4E−13	N
rs6538413	12	93 749 125	RP11-486A14.2	G	0.692	--0.09	--0.11	--0.06	1.0E−12	N
rs557536055	17	40 535 384	STAT3	C	0.701	--0.09	--0.11	--0.06	1.3E−12	N
rs60152331	1	63 177 539	RP11-230B22.1	G	0.623	--0.08	--0.10	--0.06	2.6E−12	Y
rs73369896	17	80 478 877	FOXK2	G	0.922	0.15	0.11	0.19	3.4E−12	Y
rs10811408	9	20 805 270	FOCAD	T	0.777	0.09	0.07	0.12	4.1E−12	Y
rs34817	5	102 435 260	GIN1	G	0.695	--0.08	--0.11	--0.06	5.1E−12	Y
rs71446622	13	113 365 480	ATP11A	G	0.910	0.13	0.10	0.17	5.8E−12	N
rs8138197	22	43 114 551	A4GALT	G	0.529	0.08	0.05	0.10	5.9E−12	N
rs72755040	15	64 342 757	DAPK2	G	0.945	0.17	0.12	0.21	7.1E−12	Y
rs12146644	11	95 492 878	FAM76B	A	0.618	0.08	0.06	0.10	9.6E−12	Y
rs117107603	7	149 261 825	ZNF767	C	0.984	0.30	0.21	0.39	9.6E−12	Y
rs10910476	1	234 734 956	IRF2BP2	C	0.444	0.08	0.05	0.10	1.2E−11	Y
rs1520195	3	183 736 882	ABCC5	G	0.509	--0.07	--0.10	--0.05	1.3E−11	Y
rs55693403	9	84 320 639	RP11-154D17.1	T	0.942	--0.16	--0.20	--0.11	2.4E−11	Y
rs140073759	2	152 356 937	RIF1	T	0.378	0.08	0.05	0.10	2.5E−11	Y
rs61871633	11	2 366 260	CD81-AS1	C	0.913	--0.13	--0.17	--0.09	2.7E−11	Y
rs762418439	16	691 325	AL022341.1	A	0.616	0.09	0.06	0.11	2.7E−11	Y
rs62553882	9	91 497 782	PCNPP2	C	0.945	0.16	0.11	0.21	3.9E−11	Y
rs10770059	11	9 770 910	SWAP70	T	0.351	0.08	0.05	0.10	5.3E−11	N
rs12650679	4	69 771 836	RP11-468N14.13	A	0.871	0.11	0.08	0.14	5.4E−11	Y
rs4541821	7	148 446 377	CUL1	T	0.788	0.09	0.06	0.12	5.7E−11	Y
rs117111916	19	14 529 082	DDX39A	C	0.945	--0.16	--0.20	--0.11	7.1E−11	Y
rs10916527	1	229 746 970	TAF5L	T	0.495	--0.07	--0.09	--0.05	8.1E−11	Y
rs1844428	4	145 574 196	HHIP-AS1	A	0.867	0.11	0.07	0.14	8.2E−11	Y
rs13255193	8	11 309 192	FAM167A	T	0.457	--0.07	--0.09	--0.05	1.2E−10	Y
rs759544745	7	150 759 219	SLC4A2	CGTGTGTGAGT	0.424	--0.07	--0.09	--0.05	1.6E−10	Y
rs12478953	2	71 618 599	ZNF638	T	0.339	--0.07	--0.10	--0.05	1.8E−10	Y
rs758263745	3	12 395 972	PPARG	CA	0.683	0.08	0.05	0.10	1.9E−10	Y
rs62054589	16	81 068 748	RP11-303E16.3	T	0.875	0.11	0.07	0.14	2.3E−10	Y
rs118153075	12	112 825 973	HECTD4	C	0.982	0.27	0.19	0.35	3.5E−10	N
rs2011082	6	110 734 999	DDO	G	0.222	0.08	0.06	0.11	4.1E−10	Y
rs2337113	18	46 452 327	SMAD7	A	0.536	--0.07	--0.09	--0.05	5.7E−10	Y
rs11380525	7	124 427 517	GPR37	T	0.715	--0.08	--0.10	--0.05	7.9E−10	Y
rs78587207	11	57 654 991	RP11-734C14.2	T	0.681	0.07	0.05	0.10	8.2E−10	Y
rs2140875	7	129 602 879	RP11-306G20.1	A	0.186	0.09	0.06	0.11	1.2E−09	N
rs9783086	1	225 588 376	LBR	T	0.835	--0.09	--0.12	--0.06	1.2E−09	Y
rs67795055	3	169 529 895	LRRC34	C	0.776	--0.08	--0.11	--0.05	1.6E−09	Y
rs139372052	16	67 695 483	PARD6A	T	0.995	--0.46	--0.61	--0.31	1.7E−09	Y
rs2535922	14	73 447 240	NA	A	0.609	--0.07	--0.09	--0.05	2.4E−09	Y
rs200127094	7	123 430 826	RNU6-11P	A	0.920	0.12	0.08	0.16	2.6E−09	Y
rs11072763	15	78 724 256	IREB2	A	0.222	--0.08	--0.10	--0.05	3.4E−09	Y
rs7649045	3	196 519 878	PAK2	T	0.406	0.07	0.04	0.09	7.1E−09	Y
rs2672092	15	81 870 620	CTD-2034I4.1	T	0.772	0.08	0.05	0.10	8.9E−09	Y
rs6433891	2	181 969 709	AC068196.1	G	0.299	0.07	0.05	0.09	9.7E−09	Y
rs574063085	1	118 769 284	RNA5SP56	C	0.993	--0.43	--0.57	--0.28	1.0E−08	Y
rs12514956	5	177 635 181	HNRNPAB	G	0.919	--0.12	--0.16	--0.08	1.4E−08	Y
rs111362998	15	74 760 541	UBL7-AS1	A	0.940	--0.14	--0.18	--0.09	1.5E−08	Y
rs552000109	11	85 682 778	PICALM	C	0.841	0.09	0.06	0.12	2.3E−08	Y
rs74346567	15	86 249 722	AKAP13	G	0.944	--0.13	--0.18	--0.09	3.0E−08	N
rs4446237	3	171 472 296	PLD1	C	0.534	--0.06	--0.08	--0.04	3.5E−08	Y
rs562038	1	120 254 545	PHGDH	G	0.320	0.06	0.04	0.09	4.0E−08	Y
rs117629721	10	101 786 635	snoU13	C	0.965	0.17	0.11	0.23	4.6E−08	Y
rs190629717	8	144 310 590	GPIHBP1	G	0.759	--0.07	--0.10	--0.05	4.8E−08	Y

^a^Not reported in [12, 37] or in the GWAS catalog (https://www.ebi.ac.uk/gwas/) to be associated with MCV.

### GWAS of MCV stratified by alcohol intake

Analysis of the heterogenous effects between individuals with different alcohol intakes found that no variants reached the threshold for statistical significance (*P* < 2.4 × 10^−4^). SNP rs218264 was the closest to this threshold at *P* = 5.2 × 10^−4^, although both the low and heavy drinking groups showed significant associations with this variant (Table 3). No variants reached genome-wide significance (*P* < 5 × 10^−8^) when exploring the heterogeneity of allelic effects between the different drinking groups. Specific assessment of the alcohol metabolizing pathway found no evidence of an alcohol-related association between MCV and either the *ADH* or ALDH SNPs (Supplementary Material, Table S2).

### Allele score for alcohol metabolism pathway

The genetic score used as a proxy for acetaldehyde accumulation rate/speed of clearance in drinkers only was independent of confounding factors (i.e. covariates included in multivariate model). The frequencies of the effect alleles contributing to the allele score were as follows: rs1229984_T = 0.021; rs698_T = 0.588 and rs2228093_T = 0.121. We found no evidence for an association between MCV and the allele score (*P* = 0.53). There was however evidence that the allele score was associated with alcohol intake (*P* < 2 × 10^−16^). Categorization of the allele score demonstrated that this relationship with alcohol consumption was dose-dependent (negative direction), and thus, the score can be considered valid given current knowledge of alcohol metabolism and its relationship with intake (Supplementary Material, Table S3).

### Phenome-wide analysis

We performed Phenomewide association analysis (PheWAS) to detect whether the variants implicated in MCV might impact other diseases or clinically relevant phenotypes. This showed that the SNPs contribute to a range of different diseases, with 71 SNP-outcome pairs reaching *P* < 4.8 ×10^−7^ (Supplementary Material, Table S4). The most consistent outcomes were observed for ICD-10 chapter IV codes, including disorders of mineral metabolism and disorders of lipoprotein metabolism and other lipidaemias. There was also strong evidence from three SNPs for a shared risk with neoplasms of the skin. Thyroid-related disorders were also found in two SNPs (rs2134814, rs592229), with evidence for both under- and overactive thyroid diagnoses. The G allele in rs2134814 was associated with increased MCV and hypothyroidism, while the T allele in rs592229 was associated with decreased MCV and hyperthyroidism. Other outcomes included diabetes, multiple sclerosis, hypertension, varicose veins and rheumatoid arthritis.

### Mendelian randomization

Mendelian randomization analysis demonstrated a significant causal effect of alcohol consumption on MCV. Each copy of the effect allele at rs1229984 in *ADH1B* was associated with a 0.19 decrease in drinks per week in the work by Jorgenson *et al.* (20) and was also found to reduce MCV by 0.18 femtoliters (fL) (SE = 0.002; *P* = 0.002). However, the addition of rs7686419 (*KLB*) returned a null outcome with evidence of effect heterogeneity, although the effect size of rs7686419 for drinks per week was approximately 6-fold smaller than rs1229984 (20).

**Table 3:** Summary of variants reaching nominal significance for heterogeneity of allelic effects (significance threshold: *P* < 2.1 × 10^−4^)

	Low drinkers (*n* = 228 671)				Heavy drinkers (*n* = 133 924)				
SNP	% change MCV	95%LCI	95%UCI	*P*	% change MCV	95%LCI	95%UCI	*P*	Heterogeneity *P*
rs218264	−0.50	−0.53	−0.47	4.5 × 10^−232^	−0.42	−0.45	−0.38	5.1 × 10^−104^	5.2 × 10^−4^
rs855791	−0.61	−0.64	−0.59	2.3 × 10^−462^	−0.54	−0.57	−0.51	2.3 × 10^−230^	5.9 × 10^−4^
rs144861591	−1.15	−1.20	−1.11	5.0 × 10^−487^	−1.03	−1.09	−0.97	3.8 × 10^−246^	2.0 × 10^−3^
rs4936291	0.12	0.10	0.15	7.9 × 10^−19^	0.06	0.02	0.09	7.4 × 10^−4^	4.0 × 10^−3^
rs10758657	0.41	0.38	0.44	1.4 × 10^−139^	0.34	0.30	0.38	2.9 × 10^−63^	0.01
rs243076	−0.25	−0.28	−0.23	9.4 × 10^−79^	−0.20	−0.23	−0.17	2.3 × 10^−32^	0.01
rs56142708	−0.17	−0.20	−0.15	6.5 × 10^−39^	−0.22	−0.26	−0.19	1.8 × 10^−41^	0.02
rs67971539	−0.20	−0.23	−0.17	8.4 × 10^−37^	−0.14	−0.18	−0.10	2.4 × 10^−12^	0.02
rs6592965	−0.32	−0.34	−0.29	1.7 × 10^−125^	−0.27	−0.30	−0.23	1.6 × 10^−57^	0.02
rs12650679	0.09	0.05	0.13	1.2 × 10^−5^	0.16	0.11	0.21	7.3 × 10^−11^	0.02
rs9471708	0.60	0.58	0.63	5.0 × 10^−369^	0.55	0.51	0.59	3.6 × 10^−192^	0.02
rs12582170	−0.19	−0.22	−0.15	4.7 × 10^−25^	−0.12	−0.17	−0.08	1.3 × 10^−7^	0.02
rs17296501	−0.26	−0.30	−0.23	1.3 × 10^−47^	−0.20	−0.24	−0.15	4.4 × 10^−18^	0.03
rs9801017	−0.33	−0.35	−0.30	4.1 × 10^−126^	−0.28	−0.31	−0.25	1.5 × 10^−60^	0.04
rs74401481	0.60	0.50	0.70	1.5 × 10^−32^	0.76	0.64	0.89	5.6 × 10^−34^	0.05

## Discussion

In the largest study undertaken to date, we have shown, as would be expected, that alcohol was clearly associated with an increase in MCV in a dose-dependent manner. However, the effects of alcohol on MCV were largely independent of genetic architecture, despite the association of MCV with genetic variation at 212 autosomal loci. Our analysis using Mendelian randomization provides evidence of a causal relationship between alcohol intake and MCV. However, we demonstrated a lack of association between alcohol metabolizing genes and MCV using a genetic score approach. Taken together, these findings support MCV as a marker of alcohol use disorder, although lack of specificity remains a substantial barrier in predictive accuracy and therefore clinical utility (3).

The strengths of this study are as follows: (1) the large sample size for GWAS analyses, (2) post-GWAS analysis including fixed effect inverse-variance weighted meta-analysis to generate heterogeneity statistics, (3) the use of a mixed-model approach in GWAS to account for relatedness and maximize sample size and (4) use of allele scores to explore the functional consequences of alcohol metabolizing gene variants as a proxy for acetaldehyde accumulation. There are, however, several limitations. First, the alcohol measures were based on self-report. The accuracy of self-report alcohol consumption has been questioned due to under-coverage compared with sales data (21). Second, we restricted our analysis to those of white British ancestry to limit population structure variability on the outcomes. This limits generalizability of our findings to other ethnic groups. Third, we did not undertake formal replication of findings, but our top GWAS outcomes are consistent with those reported elsewhere (11,12,15–18). Finally, we considered including folate in our models. However, folate levels were not measured in the UKB and the prevalence of folate deficiency anaemia was low (<0.002%).

The large sample size of the UKB enabled the detection of genetic variants with small effect sizes. The replication of findings in loci such as *HBS1L-MYB, TMPRSS6* and *CCND3*, which have been identified in previous GWAS for MCV (11,12,15–18), supports the validity of our outcomes. Indeed, many of the low-frequency variants with smaller effect sizes were reported in an analysis of 36 blood cell traits (11). However, we also identified 108 new loci associated with MCV providing new biological insights. We observed associations between MCV and several loci involved in DNA modification through binding and/or processing alterations (e.g. *ZNF165, TAF6, ZBTB38, ZKSCAN5*, *SPIDR*). It is known that impaired DNA synthesis delays cell division resulting in macrocytosis (22). Of the new loci identified, rs13191659 (*VN1R12P/LINC00240*) has been associated with total iron binding capacity in Hispanics (23); *DPP8* has been suggested as a candidate gene for mean corpuscular haemoglobin (MCH) in Europeans (24) and was identified as part of an LD block at 15q22.3 containing *IGDCC4-DPP8-PTPLAD1-C15orf44-SLC24A1-DENND4A* for MCH in Japanese (25); *OBFC1* and *MEGF11* have been associated with MCH but not MCV (26,27); *PAK2* has been reported to have a role in eryptosis of erythrocytes, and therefore the effect of *PAK2* on red blood cell indices might be greater than previously recognized (28); *LDB1* influences erythrocyte development by the protein product acting as a cofactor for transcription factor complexes with, for example, Gata1, Tal1, E2A and Lmo2 (29). Indeed, the critical requirement for LDB1 during early-stage erythropoiesis has been demonstrated in rodent models (30). Furthermore, several of our lead SNPs were missense variants, including rs1047891 (EAF 0.684; *P* = 1.1 × 10^−20^) (*CPS1*) alongside more well-described MCV-associated SNPs such as rs855791 (EAF 0.440; *P* = 3.0 × 10^−610^) (*TMPRSS6*) and rs3811444 (EAF 0.667; *P* = 1.1 × 10^−81^) (*TRIM58*). rs1047891 is in the 3′ untranslated section of *CPS1*, a region reported to play a key role in glycine and serum homocysteine metabolism. Allelic variation in rs1047891 has been associated with various cardiometabolic traits (31,32) and lower platelet count (33). The substitution at this SNP (T-->N; p.Thr1412Asn) increases enzymatic activity and influences nitric oxide production (34), an important mediator of vascular function. MCV has been reported to be an independent predictor for cardiovascular events (35) and rs1047891 variation is therefore a potential pathway for this relationship.

Stratification of participants by drinking status did not identify any loci that determined the effect of alcohol intake on MCV. This suggests that the pathways through which alcohol influences MCV are not mediated by genetic variation. This was supported by the causal inference for alcohol on MCV levels when using rs1229984 as a proxy for alcohol consumption in the Mendelian randomization analysis. However, the discriminatory power of MCV in identifying heavy alcohol use is modest given that alcohol accounts for only ~65% of MCV values above 100 fL (36). In addition, the turnover of erythrocytes is around 120 days meaning that recently abstinent individuals will present with evidence of alcohol consumption for several months.

Using a genetic score to define alcohol metabolism, we did not find evidence to support that acetaldehyde accumulation is important in determining MCV levels. This is contrary to the findings in Asians for MCV (37) and other alcohol-related liver function in Europeans (38). The lack of association with MCV is likely to be due to the fact that rs1229984 (ADH1B) is rare in Europeans and the ubiquitous presence of active ALDH2, the enzyme primarily involved in the rapid metabolism of acetaldehyde to acetate (39). Similar results to our own for ALDH gene polymorphisms were reported in a study of 510 white alcohol-dependent patients (40).

The PheWAS analysis showed SNP level pleiotropy for variants involved in MCV suggesting a shared genetic risk with a number of conditions. Many of these combinations have strong physiological connections with one another (e.g. mineral metabolism disorders and liver disease). The association between MCV and thyroid dysfunction is well described, with thyroid hormones being essential for erythropoiesis (41). Indeed, we found evidence to support the relationship between hypothyroidism and increased MCV (42) alongside hyperthyroidism and decreased MCV (43). Our findings suggest that some pathways, as mediated by rs2134814 (*BACH2*) and rs592229 (*SKIV2L*), convey shared genetic architecture for MCV and thyroid dysfunction. Other findings offer additional insight in areas of ongoing investigation such as the association between psoriasis and red blood cell deformability (44).

In summary, we have demonstrated that the impact of alcohol consumption on MCV is independent of allelic variation and provided new biological insights into the genetic loci determining MCV itself. The role of acetaldehyde, although likely important in determining MCV, is difficult to measure in Europeans due to rare variation in alcohol metabolizing genes. Interindividual variability in MCV in the setting of moderate to heavy alcohol consumption is likely to be due to a complex (and at present incompletely understood) interaction between genetic factors, underlying medical conditions and lifestyle factors.

## Materials and Methods

A complete description of the methods can be found in the Supplementary Material.

### UKB

The UKB is a large population cohort of ~502 000 individuals from the United Kingdom aged 40–69 years at recruitment. Only white British participants were included in this study. Ethical approval for the UKB was gained from the Research Ethics Service (reference: 17/NW/0274), and written informed consent was obtained from all participants. Analyses were conducted under approved application 15110.

### Alcohol consumption

Questions from the UKB baseline assessment were used to estimate alcohol consumption. We applied a standardized number of UK alcohol units to each drink to enable estimation of the number of units per week, as described previously (13).

### MCV measurement

Components of full blood counts were measured in UKB participants using clinical haematology analysers at the centralized processing laboratory of the UK Biocenter (Stockport, UK). Full information on the protocol can be found elsewhere (45).

### Multivariable analyses for predictors of MCV

MCV was natural log-transformed to normalize the distribution of residuals. Multivariable linear regression was applied to identify predictors of MCV. Analyses examined alcohol consumption as both a continuous and categorical predictor of MCV. All multivariable analyses were adjusted for age, sex, smoking status, history of hypothyroidism and vitamin B12 deficiency, and individuals with liver disease were removed due to the interaction between alcoholic liver disease risk and macrocytosis (7). Models were rerun with those reporting zero alcohol consumption removed.

### Genetic analyses

In July 2017, UKB released genetic information (directly typed and imputed genotypes) for 487 406 individuals to approved collaborators. Genotyping, quality control and imputation were performed centrally by UKB and have been described previously (46).

#### GWAS analysis

Autosomal genetic association analysis was conducted for ln(MCV) using a linear mixed model in BOLT-LMM v2.3.4 (47), adjusted for genotyping array and covariates outlined in multivariable analyses plus alcohol consumption in units/week as a continuous variable. Distance-based clumping was used for defining loci. Genomic control adjustments were applied for standard errors and *P*-values.

#### Heterogeneity of allelic effects by drinking group

Variants reaching *P* < 5 × 10^−8^ and surviving distance-based clumping (i.e. lead SNPs) were explored for heterogeneous outcomes based on drinking category. GWAMA was used to run a fixed effect inverse-variance weighted meta-analysis on outcomes and generate heterogeneity statistics for allelic effects between groups, which is equivalent to fitting an interaction term (48). Any variant reaching the Bonferroni-corrected threshold (*P* < 0.05/‘number of lead SNPs from unstratified GWAS’) was considered statistically significant.

#### MCV heritability

To characterize the heritability of MCV, we applied single-trait LD­score regression through LD Hub v1.9.3 (http://ldsc.broadinstitute.org/ldhub/) (49).

#### Phenome­wide association analysis

Gene ATLAS (http://geneatlas.roslin.ed.ac.uk/) was used as a lookup for outcomes from PheWAS analysis performed on UKB traits (50).

#### Impact of genetic score for acetaldehyde on MCV

To test the assumption that acetaldehyde is important in MCV, we used genotype data for SNPs in *ADH1B*, *ADH1C* and *ALDH1B* to construct a genetic score. The SNPs rs1229984 (*ADH1B*), rs698 (*ADH1C*) and rs2228093 (*ALDH1B*) were used to generate an unweighted allele score based on number of *ADH* alleles increasing the metabolism of ethanol to acetaldehyde and the number of *ALDH* alleles slowing the metabolism of acetaldehyde to acetate. This score (0–6) was used as a continuous predictor alongside covariates previously outlined in multivariable analyses. The selected variants were independent (*r*^2^ < 0.01 for all SNP pairs).

#### Mendelian randomization

MR-Base v0.4.21 was used for performing Mendelian randomization to explore the causal relationship between alcohol consumption and MCV (51). The causal estimates between exposure and outcome were obtained using the two-sample Mendelian randomization inverse variance-weighted method.

Results are reported using STROBE guidelines. A checklist can be found in the Supplementary Material.

## Supplementary Material

STROBE_checklist_ddab147

Supplementary_material-HMG_ddab147

## Data sharing statement

All data needed to evaluate the conclusions in the paper are present in the paper and/or the Supplementary Materials. The data from UKB were provided under license by UKB, who is the owner of the data. Requests for access to the data should be directed to UKB as per the material transfer agreement. Additional data related to this paper may be requested from the authors.
