# Supplementary material for: Assessing the impact of alcohol consumption on the genetic contribution to mean corpuscular volume
Source: Hum Mol Genet. 2021 Jun 8;30(21):2040–51. doi: 10.1093/hmg/ddab147 (PMC8522631; doi:10.1093/hmg/ddab147)
Supplement: Supplementary_material-HMG_ddab147 [file supplementary_material-hmg_ddab147.docx]

**SUPPLEMENTARY MATERIAL**

**Assessing the impact of alcohol consumption on the genetic contribution to mean corpuscular volume**

Andrew Thompson, Katharine King, Andrew P Morris, Munir Pirmohamed

**METHODS**

**UK Biobank**

The UK Biobank is a large population cohort of ~502,000 individuals from the United Kingdom aged 40 to 69 years at the time of recruitment. Individuals with contact information available via National Health Service central registers and who met the age and distance to recruitment centre criteria were invited by letter to join the study (~9 million people). Baseline assessment was undertaken at one of 22 centres across the United Kingdom between 2006 and 2010. Each participant completed a comprehensive demographic, lifestyle, and health questionnaire, underwent clinical phenotyping, provided biological samples (i.e., blood, urine, and saliva), and agreed to have their health record accessed for baseline and follow-up outcomes [1]. Only white British participants were included in this study, defined based on self-reported ethnicity and genetic data. Ethical approval for the UK Biobank was gained from the Research Ethics Service (REC reference: 16/NW/0274), and written informed consent was obtained from all participants. The current analyses were conducted under approved UK Biobank data application number 15110.

**Calculating alcohol consumption**

Questions from the UK Biobank baseline assessment were used to estimate alcohol consumption. All participants that indicated they consumed alcohol were asked to quantify their intake per week or per month using standard drink sizes (e.g. "In an average WEEK, how many glasses of RED wine would you drink? [There are six glasses in an average bottle]"); pictures accompanied these questions to provide visual representation of each measure. We then applied a standardised number of UK alcohol units to each drink to enable estimation of the number of units per week, as described previously [2]. Non-drinkers were assigned a value of zero units/week, and those that refused information about their drinking were excluded.

**MCV measurement**

Components of full blood counts were measured in UK Biobank participants using clinical haematology analysers at the centralized processing laboratory of the UK Biocenter (Stockport, UK). Research blood samples for the baseline assays of UK Biobank volunteers were collected into 4 ml EDTA vacutainers by vacuum draw at the UK Biobank assessment centres and stored at 4 °C. The samples were transported to the UK Biocenter in temperature-controlled shipping boxes. Subsequent processing was undertaken using four Beckman Coulter LH700 Series instruments. The analyser measures full blood counts by a combination of fluorescence and impedance flow cytometry. Full information on the protocol can be found elsewhere [3].

**Multivariable analyses for predictors of MCV**

MCV was natural log-transformed to normalise the distribution of residuals. Multivariable linear regression was applied to identify predictors of MCV. Analyses examined alcohol consumption as both a continuous variable and in categories of zero, light (1-14 units per week), moderate (15-34 in women and 15-49 in men) and heavy (≥35 in women and ≥50 in men) consumption for predicting MCV. All multivariable analyses were adjusted for age, sex, smoking status, history of hypothyroidism and vitamin B12 deficiency, and individuals with liver disease were removed. Multicollinearity was explored using the variance inflation factor. Models were rerun with those reporting zero alcohol consumption removed to account for previous drinkers becoming teetotal due to, for example, medical reasons.

**Genetic analyses**

In July 2017, UK Biobank released genetic information (directly typed and imputed genotypes) for 487,406 individuals to approved collaborators. Most (90%) of the participants were genotyped on the UK Biobank Axiom Array, with the remaining 10% genotyped on the Affymetrix UK BiLEVE Axiom Array. There is >95% content overlap between the arrays. Genotyping, quality control, and imputation were performed centrally by UK Biobank, and has been described previously [4]. Imputation was performed up to combined reference panels from the 1000 Genomes Project (Phase 3), UK10K, and the Haplotype Reference Consortium [5].

*GWAS analysis*

Autosomal genetic association analysis was conducted for ln(MCV) using a linear mixed model in BOLT-LMM v2.3.4 [6], adjusted for genotyping array and covariates outlined in multivariable analyses plus alcohol consumption in units/week as a continuous variable. The BOLT-LMM model includes a random effect derived from a genetic relationship matrix to account for population structure and relatedness. Filtering of variants was undertaken based on imputation quality (INFO ≥0.4) and minor allele frequency of 0.005. Distance-based clumping was used for defining loci, such that genome-wide significant (*P* < 5 × 10^–8^) SNPs were ranked from most significant to least significant and were retained if they did not map +/- 500kb of a more significant SNP. Genomic control adjustments were applied for standard errors and p-values.

*Heterogeneity of allelic effects by drinking group*

Variants reaching *P* < 5 × 10^–8^ and surviving distance-based clumping (i.e. lead SNPs) were explored for heterogeneous outcomes based on drinking category. As such, drinking status was collapsed further to a binary outcome (i.e. low drinkers [zero and light] and high drinkers [moderate and heavy]) and stratified GWAS performed as above, but without alcohol consumption as a covariate. GWAMA was used to run a fixed effect inverse-variance weighted meta-analysis on outcomes and generate heterogeneity statistics for allelic effects between groups, which is equivalent to fitting an interaction term [7]. Any variant reaching the Bonferroni corrected threshold (P < 0.05 / “*number of lead SNPs from unstratified GWAS*”) was considered statistically significant. An alternative genome-wide approach for exploring heterogeneity of allelic effects was also used, such that any variant with a heterogeneity statistic *P* < 5 × 10^–8^ was considered significant; this was independent of the unstratified GWAS outcomes.

*MCV* heritability

To characterize the heritability of MCV, we applied single-trait LD­score regression through LD Hub v1.9.3 (http://ldsc.broadinstitute.org/ldhub/) [8]. The LD scores for each of the ~1.2 million SNPs used in the regression model are calculated using individuals of European ancestry from the 1000 Genomes Project.

*Phenome­wide association analysis (PheWAS)*

Gene ATLAS (http://geneatlas.roslin.ed.ac.uk/) was used as a lookup for outcomes from PheWAS analysis performed on UK Biobank traits [9]. Associations have been computed using linear mixed models through the DISSECT software. The database contains data from >452,000 white British individuals, >30 million variants, and 778 traits; we only considered phenotypes defined by ICD-10 codes (n = 496) [10]. All variants obtaining genome-wide significance in the unstratified GWAS were explored. This information was used to derive a phenome-wide significance threshold, divided by the number of independent tests, i.e., P < 4.8 x10^-7^ [0.05 / (496 * “*number of lead SNPs from unstratified GWAS”*)].

*Impact of genetic score for acetaldehyde on MCV*

To test the assumption that acetaldehyde is important in MCV, we used genotype data for SNPs in *ADH1B*, *ADH1C* and *ALDH1B* to construct a genetic score. The major pathway of alcohol metabolism involves two steps; first, oxidation of ethanol to acetaldehyde primarily by alcohol dehydrogenases (*ADHs*); and second, rapid oxidation of acetaldehyde to acetate by aldehyde dehydrogenases (*ALDHs*). Variation in the isoforms of *ADH* and *ALDH* have been shown to influence the rate of this reaction, ultimately changing acetaldehyde accumulation. SNP selection was based on previous evidence relating to alcohol metabolism kinetics and allele frequencies in European ancestry populations [11, 12]. The SNPs rs1229984 (*ADH1B*), rs698 (*ADH1C*) and rs2228093 (*ALDH1B*) were used to generate an unweighted allele score based on number of *ADH* alleles increasing the metabolism of ethanol to acetaldehyde and the number of *ALDH* alleles slowing the metabolism of acetaldehyde to acetate. This score (0-6) was used as a continuous predictor alongside covariates previously outlined in multivariable analyses. Only those that consume alcohol were included in this analysis given that ethanol is essential to acetaldehyde accumulation in this context. Linear and logistic regression was used to check the assumption that genetic variants under study were not associated with confounding factors.

*Mendelian randomization*

MR-Base v0.4.21 was used for performing Mendelian randomization to explore the causal relationship between alcohol consumption and MCV [13]. The exposure was alcoholic drinks per week, with the missense variant rs1229984 in *ADH1B* being used as the instrumental variable. The effect of allele A in non-Hispanic white individuals has been estimated as a 0.19 decrease in drinks per week in previous work [14]. The outcome of MCV was taken from investigations using UK Biobank with the data standardised using SD [15]. Harmonisation between exposure data and outcome data was undertaken to ensure effects corresponded to the same allele. A sensitivity analysis was performed by adding rs7686419 as an additional instrumental variable, with the effect size again taken from the work by Jorgenson and colleagues [14]. This variant is upstream of *KLB*, which has been associated with alcohol consumption in several large GWAS analyses [2, 14, 16, 17]. The causal estimates between exposure and outcome was obtained using the two-sample Mendelian randomization inverse variance–weighted method.

Analyses conducted using R V3.5.2 [18], unless stated otherwise.

**Table S1:** Summary of linear and logistic regression models with only drinkers (n=280,360)

| **Risk Factor** |  | **Change in MCV (%)** | **95% CI** | ***P*** |
| --- | --- | --- | --- | --- |
| Alcohol: continuous variable |  |  |  |  |
| Alcohol (5 units) |  | 0.28 | 0.27 to 0.28 | <1.0 x 10^-320^ |
| Sex (Ref: Female) |  | -0.40 | -0.44 to -0.37 | 3.9 x 10^-104^ |
| Age at recruitment |  | 0.06 | 0.06 to 0.07 | <1.0 x 10^-320^ |
| Never Smoker (Ref: current) |  | -2.00 | -2.06 to -1.94 | <1.0 x 10^-320^ |
| Previous smoker (Ref: current) |  | -1.88 | -1.94 to -1.82 | <<1.0 x 10^-320^ |
| Hypothyroidism |  | -0.14 | -0.23 to -0.05 | 3.0 x 10^-3^ |
| B12 deficiency |  | 0.40 | -0.00 to 0.80 | 0.05 |
|  |  |  |  |  |
| Alcohol: categorical variable |  |  |  |  |
| Drinking status (Ref: Light)  Moderate  Heavy |  | 1.20  2.87 | 1.16 to 1.23  2.80 to 2.94 | <1.0 x 10^-320^  <1.0 x 10^-320^ |
| Sex (Ref: Female) |  | -0.24 | -0.27 to -0.20 | 4.1 x 10^-38^ |
| Age at recruitment |  | 0.06 | 0.06 to 0.07 | <1.0 x 10^-320^ |
| Never Smoker (Ref: current) |  | -2.08 | -2.14 to -2.02 | <1.0 x 10^-320^ |
| Previous smoker (Ref: current) |  | -1.93 | -1.99 to -1.87 | <1.0 x 10^-320^ |
| Hypothyroidism |  | -0.15 | -0.24 to -0.06 | 1.6 x 10^-3^ |
| B12 deficiency |  | 0.42 | 0.02 to 0.83 | 0.04 |

**Table S2.** Summary of heterogeneity of allelic effects for selected SNPs in alcohol metabolising genes.

| SNP | Locus | Effect allele | Effect allele direction | Light drinkers % change | Heavy drinkers % change | Heterogeneity *P* |
| --- | --- | --- | --- | --- | --- | --- |
| rs1229984 | ADH1B | T | -- | -0.018 | -0.089 | 0.36 |
| rs698 | ADH1C | T | -- | -0.025 | -0.007 | 0.41 |
| rs2228093 | ALDH1B | C | +- | 0.028 | -0.032 | 0.06 |

| Allele score | N | B (units/week) | SE | *P* |
| --- | --- | --- | --- | --- |
| 0 | 36695 | REF | REF | REF |
| 1 | 108940 | -0.22 | 0.09 | 0.01 |
| 2 | 99759 | -0.53 | 0.09 | 3.1 x 10^-9^ |
| 3 | 27466 | -1.35 | 0.12 | <2.0 x 10^-16^ |
| ≥4 | 3169 | -2.42 | 0.27 | <2.0 x 10^-16^ |

**Table S3**. Effect of allele score from alcohol metabolising genes on alcohol consumption

**Table S4**. Variant-trait significant outcomes from PheWAS.

| **Variant** | **Locus** | **Trait** | **p-value** |
| --- | --- | --- | --- |
| rs10770059 |  | I10-I15 Hypertensive diseases | 4.61E-10 |
|  |  | I10 Essential (primary) hypertension | 1.01E-09 |
|  |  |  |  |
| rs10901252 |  | I80 Phlebitis and thrombophlebitis | 7.10E-18 |
|  |  | I84 Haemorrhoids | 1.36E-08 |
|  |  |  |  |
| rs115447786 |  | N40-N51 Diseases of male genital organs | 3.25E-07 |
|  |  | I10 Essential (primary) hypertension | 3.66E-07 |
|  |  |  |  |
| rs12193223 |  | E83 Disorders of mineral metabolism | 4.38E-44 |
|  |  |  |  |
| rs12582170 |  | N43 Hydrocele and spermatocele | 4.06E-08 |
|  |  |  |  |
| rs13191659 |  | E83 Disorders of mineral metabolism | 6.61E-195 |
|  |  |  |  |
| rs13194984 |  | E83 Disorders of mineral metabolism | 1.81E-131 |
|  |  | I83 Varicose veins of lower extremities | 2.68E-08 |
|  |  |  |  |
| rs13255193 |  | I10 Essential (primary) hypertension | 6.1E-08 |
|  |  | I10-I15 Hypertensive diseases | 6.9E-08 |
|  |  |  |  |
| rs144861591 |  | E83 Disorders of mineral metabolism | < 1.0E-300 |
|  |  | E70-E90 Metabolic disorders | 3.64E-16 |
|  |  | K74 Fibrosis and cirrhosis of liver | 8.64E-09 |
|  |  | D75 Other diseases of blood and blood-forming organs | 3.13E-07 |
|  |  |  |  |
| rs147493146 |  | E83 Disorders of mineral metabolism | 4.62E-125 |
|  |  |  |  |
| rs149359690 |  | E83 Disorders of mineral metabolism | 2.24E-253 |
|  |  | E70-E90 Metabolic disorders | 6.90E-12 |
|  |  | D60-D64 Aplastic and other anaemias | 2.88E-07 |
|  |  |  |  |
| rs174567 |  | K80 Cholelithiasis | 7.93E-09 |
|  |  | K80-K87 Disorders of gallbladder, biliary tract and pancreas | 9.03E-09 |
|  |  | J45 Asthma | 9.04E-09 |
|  |  | J40-J47 Chronic lower respiratory diseases | 3.90E-08 |
|  |  |  |  |
| rs2057726 |  | E83 Disorders of mineral metabolism | 5.73E-26 |
|  |  | L40 Psoriasis | 2.04E-15 |
|  |  | L40-L45 Papulosquamous disorders | 5.59E-12 |
|  |  | I83 Varicose veins of lower extremities | 5.69E-10 |
|  |  |  |  |
| rs2134814 |  | J45 Asthma | 3.13E-15 |
|  |  | E03 Other hypothyroidism | 5.83E-15 |
|  |  | J40-J47 Chronic lower respiratory diseases | 1.08E-14 |
|  |  | E00-E07 Disorders of thyroid gland | 6.97E-13 |
|  |  | J33 Nasal polyp | 1.27E-11 |
|  |  | C44 Other malignant neoplasms of skin | 3.75E-09 |
|  |  | C43-C44 Melanoma and other malignant neoplasms of skin | 1.10E-08 |
|  |  |  |  |
| rs2337113 |  | K63 Other diseases of intestine | 1.36E-17 |
|  |  | C15-C26 Malignant neoplasms of digestive organs | 7.23E-09 |
|  |  | K55-K64 Other diseases of intestines | 3.30E-07 |
|  |  |  |  |
| rs243076 |  | E10-E14 Diabetes mellitus | 3.19E-08 |
|  |  | E11 Non-insulin-dependent diabetes mellitus | 7.67E-08 |
|  |  |  |  |
| rs362538 |  | E83 Disorders of mineral metabolism | 1.06E-69 |
|  |  | G35 Multiple sclerosis | 3.55E-10 |
|  |  | G35-G37 Demyelinating diseases of the central nervous system | 7.70E-09 |
|  |  | I83 Varicose veins of lower extremities | 4.11E-07 |
|  |  |  |  |
| rs592229 |  | K90 Intestinal malabsorption | 1.02E-149 |
|  |  | G35 Multiple sclerosis | 1.45E-25 |
|  |  | G35-G37 Demyelinating diseases of the central nervous system | 2.68E-25 |
|  |  | K90-K93 Other diseases of the digestive system | 1.25E-24 |
|  |  | L40 Psoriasis | 2.12E-17 |
|  |  | L40-L45 Papulosquamous disorders | 3.42E-16 |
|  |  | M06 Other rheumatoid arthritis | 6.41E-12 |
|  |  | M05 Seropositive rheumatoid arthritis | 8.77E-09 |
|  |  | I10-I15 Hypertensive diseases | 1.87E-08 |
|  |  | I10 Essential (primary) hypertension | 1.97E-08 |
|  |  | E05 Thyrotoxicosis [hyperthyroidism] | 5.80E-08 |
|  |  | E78 Disorders of lipoprotein metabolism and other lipidaemias | 8.09E-08 |
|  |  |  |  |
| rs6440006 |  | N40-N51 Diseases of male genital organs | 3.72E-07 |
|  |  |  |  |
| rs7089063 |  | K80 Cholelithiasis | 8.44E-10 |
|  |  | K80-K87 Disorders of gallbladder, biliary tract and pancreas | 1.54E-07 |
|  |  |  |  |
| rs7705526 |  | L82 Seborrhoeic keratosis | 7.52E-09 |
|  |  | C43-C44 Melanoma and other malignant neoplasms of skin | 1.02E-08 |
|  |  | C44 Other malignant neoplasms of skin | 5.33E-08 |
|  |  | D37-D48 Neoplasms of uncertain or unknown behaviour | 4.08E-07 |
|  |  |  |  |
| rs78378222 |  | C43-C44 Melanoma and other malignant neoplasms of skin | 8.72E-17 |
|  |  | C44 Other malignant neoplasms of skin | 1.94E-15 |
|  |  | L80-L99 Other disorders of the skin and subcutaneous tissue | 1.59E-08 |
|  |  |  |  |
| rs855791 |  | D60-D64 Aplastic and other anaemias | 4.96E-11 |
|  |  | D64 Other anaemias | 7.13E-10 |
|  |  |  |  |
| rs964184 |  | E78 Disorders of lipoprotein metabolism and other lipidaemias | 2.45E-31 |
|  |  | E70-E90 Metabolic disorders | 9.09E-22 |

**REFERENCES**

1. Sudlow, C., et al., *UK biobank: an open access resource for identifying the causes of a wide range of complex diseases of middle and old age.* PLoS Medicine, 2015. **12**(3).

2. Thompson, A., et al., *Functional validity, role, and implications of heavy alcohol consumption genetic loci.* Science Advances, 2020. **6**(3): p. eaay5034.

3. Sheard, S., R. Nicholls, and J. Froggat, *UK Biobank Haematology Data Companion Document V1.9 [accessed 19 Sep 2019].* 2017.

4. Bycroft, C., et al., *Genome-wide genetic data on~ 500,000 UK Biobank participants.* BioRxiv, 2017: p. 166298.

5. Huang, J., et al., *Improved imputation of low-frequency and rare variants using the UK10K haplotype reference panel.* Nature Communications, 2015. **6**(1): p. 1-9.

6. Loh, P.-R., et al., *Efficient Bayesian mixed-model analysis increases association power in large cohorts.* Nature Genetics, 2015. **47**(3): p. 284-290.

7. Mägi, R. and A.P. Morris, *GWAMA: software for genome-wide association meta-analysis.* BMC Bioinformatics, 2010. **11**(1): p. 288.

8. Zheng, J., et al., *LD Hub: a centralized database and web interface to perform LD score regression that maximizes the potential of summary level GWAS data for SNP heritability and genetic correlation analysis.* Bioinformatics, 2017. **33**(2): p. 272-279.

9. Canela-Xandri, O., K. Rawlik, and A. Tenesa, *An atlas of genetic associations in UK Biobank.* Nature Genetics, 2018. **50**(11): p. 1593-1599.

10. Canela-Xandri, O., et al., *A new tool called DISSECT for analysing large genomic data sets using a Big Data approach.* Nature Communications, 2015. **6**(1): p. 1-6.

11. Birley, A.J., et al., *ADH single nucleotide polymorphism associations with alcohol metabolism in vivo.* Human Molecular Genetics, 2009. **18**(8): p. 1533-1542.

12. Edenberg, H.J. and J.N. McClintick, *Alcohol dehydrogenases, aldehyde dehydrogenases, and alcohol use disorders: a critical review.* Alcoholism: Clinical and Experimental Research, 2018. **42**(12): p. 2281-2297.

13. Hemani, G., et al., *The MR-Base platform supports systematic causal inference across the human phenome.* Elife, 2018. **7**: p. e34408.

14. Jorgenson, E., et al., *Genetic contributors to variation in alcohol consumption vary by race/ethnicity in a large multi-ethnic genome-wide association study.* Molecular Psychiatry, 2017. **22**(9): p. 1359-1367.

15. Astle, W.J., et al., *The allelic landscape of human blood cell trait variation and links to common complex disease.* Cell, 2016. **167**(5): p. 1415-1429. e19.

16. Sanchez-Roige, S., et al., *Genome-wide association study meta-analysis of the Alcohol Use Disorders Identification Test (AUDIT) in two population-based cohorts.* American Journal of Psychiatry, 2019. **176**(2): p. 107-118.

17. Schumann, G., et al., *KLB is associated with alcohol drinking, and its gene product β-Klotho is necessary for FGF21 regulation of alcohol preference.* Proceedings of the National Academy of Sciences, 2016. **113**(50): p. 14372-14377.

18. Team, R.C., *R: A language and environment for statistical computing.* 2013.
